# Class III Treatment with Skeletal and Dental Anchorage: A Review of Comparative Effects

**DOI:** 10.1155/2018/7946019

**Published:** 2018-07-02

**Authors:** Roberta Clemente, Luca Contardo, Christian Greco, Roberto Di Lenarda, Giuseppe Perinetti

**Affiliations:** ^1^Department of Medical, Surgical and Health Sciences, School of Dentistry, University of Trieste, Trieste, Italy; ^2^Azienda Sanitaria dell'Alto Adige, Ospedale di Merano, Bolzano, Italy; ^3^Private Practice, Nocciano (PE), Italy

## Abstract

**Objectives:**

This review addresses the comparative effects of skeletal anchored maxillary protraction (MP) versus dental anchored MP.

**Materials and Methods:**

The studies retrieved had to have both test and control groups treated by the use of a facemask with or without the use of skeletal anchorage though either (palatal/buccal) maxillary or mandibular miniscrews/miniplates, respectively.

**Results:**

Nine articles were included. Dentoalveolar changes were seen in all the studies. In particular, a significant proclination of the upper incisors was documented in the group treated with a dental anchorage facial mask, as compared to that treated with skeletal anchorage. Comparing the two methods, almost all the studies indicated a greater maxillary advancement in the group treated with skeletal anchorage.

**Conclusions:**

Therapies with skeletal anchorage produce greater maxillary protraction, reducing undesirable dental effects.

## 1. Introduction

Skeletal Class III malocclusion is one of the most arduous malocclusions to treat in orthodontics. It can be caused by a retrognathic maxilla, a prognathic mandible, or a combination of both [1, 2]. The facemask (FM) is the common appliance for the treatment of skeletal Class III patients with maxillary retrusion, as it stimulates maxillary advancement and prevents mandibular development [3]. One of the limitations in maxillary protraction with a conventional tooth-borne type appliance is the loss of dental anchorage, especially in the late mixed or permanent dentition phases [4, 5]. Several studies reported that the use of a facemask in combination with tooth-borne anchorage appliances induces the following skeletal and dental changes: forward movement of the maxilla, downward and backward rotation of the mandible, closing rotation of the palatal plane, proclination of the maxillary incisors, mesialization and extrusion of the maxillary molars, and lingualization of the mandibular incisors [6, 7]. Since the skeletal effect decreases and the dental effect increases with age [8, 9], some clinicians and researchers introduced skeletal anchorage treatments, such as intentionally ankylosed deciduous canines [10], onplants [11], osseointegrated implants [12], orthodontic miniscrews [13], and most recently miniplates [14–16] to maximize the skeletal effects of the protraction and prevent the undesirable dental effects. However, skeletal anchorage devices show certain disadvantages, they require surgical invasive procedures to insert and remove them, and some of the components may not be stable during the treatment [17].

The aim of this narrative review is to examine the effectiveness of interceptive treatment of Class III malocclusions using skeletal anchorage and to verify if the treatments with temporary anchored devices (TADs) produce greater maxillary advancement than tooth-anchored maxillary protraction and/or reduce some dental side effects. Since orthopedic treatment of Class III malocclusion often involves maxillary expansion with beneficial effects [18] or even associated pain [19], herein, studies reporting a combinational use of FM and maxillary expansion were also included.

## 2. Materials and Methods

### 2.1. Search Strategy

Articles were identified through a literature survey carried out as a result of the following databases: (1) PubMed, (2) SCOPUS, (3) Latin American and Caribbean Health Sciences (LILACS), (4) Scientific Electronic Library Online (SciELO), and (5) the Cochrane Library. The survey covered the period from inceptions to the last access on 01 November 2017 with no language restrictions. The eligibility assessment was performed independently by two blinded authors. A third author was consulted if necessary.

### 2.2. Eligibility Criteria

The studies retrieved had to be RCTs or either prospective or retrospective CCTs. They had to include healthy growing subjects treated for skeletal Class III malocclusion by the use of a facemask, with or without maxillary expansion, in the control group, and by the use of skeletal anchorage though either (palatal/buccal) maxillary or mandibular miniscrews/miniplates in the experimental group. Studies without a control group or with the untreated group were excluded. Publications such as case reports, case series, reviews, and opinion articles were excluded.

### 2.3. Data Items

The following data items were extracted: study design, prospective or retrospective enrolment, sample size, gender distribution, age, Class III description, type of appliance used, indicators of skeletal maturity and distribution of subjects according to growth phase, treatment duration, and when treatment was stopped. Regarding the treatment effects, the following items were also collected: skeletal, dentoalveolar, and soft tissues effects and authors' conclusions on the treatment efficiency. Forms used for data extraction were mostly predefined at the protocol stage by two authors.

### 2.4. Quality of the Studies

Although this is not a systematic review, a quality analysis of the included studies was performed. As no single approach in assessing methodologic soundness may be appropriate for all reviews [29, 30], a dedicated evaluation of the quality in individual studies (performed independently by two expert authors) was used that followed preestablished characteristics, along with the scores that were assigned to the individual retrieved articles detailed in Table 1. The quality of the studies, with a maximum possible score of 16, was considered as follows:

- Low: total score ≤ 7 points
- Medium: total score > 7 and ≤ 10 points
- Medium/high: total score > 10 and ≤ 14 points
- High: total score > 14 points.

## 3. Results

### 3.1. Study Designs

A total of 9 studies [20–28] were judged eligible for inclusion and are listed in Table 2. Publications years are included between 2010 [21] and 2016 [20, 28]. Only four studies were prospective [20, 21, 23, 27], other four were retrospective [24–26, 28], and, finally, only one was a randomized clinical trial [22]. The sample sizes ranged from a minimum of 20 subjects [25, 28] to a maximum of 60 ones [26] with an average of 44.5 and, overall, there is not a clear difference between sex, even if there is a prevalence of treated female subjects; only one study [28] did not specify the gender of the treated group. The age of patients at the beginning of therapy was between 8.1 ± 1.5 years and 11.9 ± 1.8 years [23]. Regarding the diagnosis of Class III malocclusion, the parameters used were the angle ANB [20, 22, 25–28], Wits [20–23, 26, 27], anterior cross-bite or a head-to-head relationship, used in almost all studies, and, finally, a Class III molar relationship [21, 23, 26, 27]. One study [24] did not use skeletal parameters but they adopted negative overjet as the only parameter. In two studies skeletal maturation was assessed at the first stage of treatment by hand and wrist radiography [20, 27]. In the first study [20], all the patients were in a prepubertal growth phase, while in the second one [27] the subjects were in a phase ranging from prepubertal to peak pubertal growth. The remaining studies [21–25, 28] used the CVM method. In four studies [21–23, 28] all subjects were in a phase between CS1 and CS3; in one study [24] the treatment was performed during the growth peak (CS3-CS4); in another study [25] some patients were in a prepubertal phase, and others in the pubertal phase (CS2- CS4). Only one study [26] did not specify the growth period in which the treatment was carried out. Regarding the type of treatment performed, the subjects of the control group of all studies used a therapy with rapid palatal expander and facemask; only one study [20] used bite plate instead of RPE. Regarding the group treated with skeletal anchorage, various methods have been used. Three studies [20, 21, 23] used the bone-anchored maxillary protraction (BAMP) method, placing mandibular miniplates between the lateral incisor and the canine and fixing them with 2 miniscrews and, in the maxillary, miniscrews were inserted between the second premolar and the first molar [20] or 2 miniplates in the infrazigomatic buttress fixed with 3 miniscrews [21, 23]. Class III elastics were applied with a force of 200g [20] or 250g [21, 23] per side. In one study [22], they placed miniscrews in the maxillary zygomatic process to anchor the traction for the mask with a force of 250g per side. Another study [25] used a skeletal anchored system by positioning the miniplates at the zygomatic process, fixing them with 3 miniscrews and tractioning the maxilla with the facemask with a force of 400g per side. Two studies [27, 28], instead, placed the miniplates laterally to the pyriform aperture of the nasal walls and they were fixed with miniscrews; the traction was exerted through a facial mask with a force of 400g per side. One study [26] used a hybrid Hyrax expander by placing 2 miniscrews in the anterior palate and a rapid expander was connected to the miniscrews and the first permanent molars. Two arms were also welded to the expander for the positioning of the elastics connected to the mask and to exert a traction of 380g per side. Finally, in the last study [24], miniplates and traction with a face mask were used but the precise site in which they were positioned was not specified. All treatments finished with the achievement of a positive overjet [20–23], > 2mm [25] or > 4mm [27, 28]; in two studies [24, 26] it was not specified. In some cases [21, 23, 25] an overcorrection has been reached up to the second molar class.

### 3.2. Main Results

Main results in the included studies are summarized in Table 3. Dentoalveolar changes were seen in all the studies of this review. In particular, a significant proclination of the upper incisors was documented in the group treated with a simple facial mask compared to that treated with skeletal anchorage in six studies [20, 22, 25–28]. Regarding lower incisors, however, there were conflicting results. In subjects treated with facemask, both in the dental anchorage and in the skeletal anchorage groups, a more or less pronounced lingualization of the lower incisors was found in three studies [22, 27, 28]. In two studies [20, 21], in the group treated with mandibular miniplates and Class III elastics, a proclination of the lower incisors was detected. Furthermore, two studies [26, 28] showed a greater mesialization of the upper molars in the group treated with RPE + FM. At skeletal level, all studies show a greater maxillary advancement in subjects treated with skeletal anchored, with the exception of two studies [22, 26], where there was no significant difference between the groups.

In the study of Aglarci et al. [20], maxillary anterior displacement was evident in both treatment groups. Further, double the amount of maxillary advancement was achieved in the SA group (A-y: 2.72 ± 1.69 mm, Co-A: 3.42 ± 2.12 mm) as compared to the FM group (A-y: 1.11 ± 1.44 mm, Co-A: 2.54 ± 3.17 mm). Both the treatment methods conducted in the study [20] prevented the advancement of mandibular prognathism (Co-Gn; FM: 2.51 ± 2.52 mm, SA: 2.25 ± 2.47 mm).

Cevidanes et al. [21] found that the BAMP protocol produced significantly larger maxillary advancement than the RME/ FM therapy (Co-A; FM: 2.4 ± 1.4 mm, BAMP: 5.3 ± 2.0 mm); mandibular sagittal changes were similar (Co-Gn; FM: 1.5 ± 1.6 mm, BAMP 2.1 ± 1.7 mm).

In the study of Ge [22], Co-A showed average improvements of 4.93 mm in the MS/FM group and 5.04 mm in the FM group, and A to Nperp increased 3.37 mm and 2.53 mm, respectively. The skeletal changes in the maxilla displacement were on average 2.6 mm in the RME/FM group and 3.7 mm in the BAMP group in the study of Hino et al. [23].

Koh et al. [24] indicated an increase in maxillary length (Co-A) of 3.05 ± 1.93 mm in the FM group and 4.60 ± 2.04 mm in the SA group, stating that this difference is much more evident in patients in CS3 compared to those in CS4 (FM/CS3: 3.95 ± 1.14 mm, SA/CS3: 5.82 ± 1.62 mm; FM/CS4: 2.01 ± 2.16 mm, SA/CS4: 2.92 ± 2.55 mm). Mandibular sagittal changes (Co-Gn) were 2.33 ± 1.70 mm and 3.04 ± 3.18 mm in the FM and SA group respectively.

Lee et al. [25] stated a forward movement of point A (A to Nperp) of 3.18 ± 1.79 mm in the MP/FM group and 1.44 ± 1.44 mm in the FM group. In terms of the anterior-posterior position of the mandible, both the MP/FM and FM groups had a posterior repositioning of the mandible (Pog to Nperp: -1.45 ± 1.71 mm and -3.78 ± 3.06 mm).

Ngan et al. [26] declared similar results in the two groups; OLp (occlusal plane perpendicular), A point moved forward, 0.72 ± 1.29 mm and 0.74 ± 1.23 mm in the FM group and SA group, respectively. Also, mandibular changes were similar (OLp-Pg; FM -2.28 ± 1.43 mm, SA -2.31 ± 2.15 mm).

Sar et al. [27] described the more forward movement of the maxilla in the MP/FM group than in the FM group (A-VR 2.83 ± 0.93 mm, Co-A 3.26 ± 1.82 mm and A-VR 2.16 ± 1.38 mm, Co-A 1.80 ± 1.70 mm, respectively). Regarding the mandibular skeletal measurements, the mandible was positioned backward significantly in both treatment groups (Pg-VR -2.53 ± 2.10 mm, Co-Gn -0.30 ± 2.15 mm in the MP/FM group, Pg-VR -3.36 ± 2.51, Co-Gn 0.43 ± 2.15 mm in the FM group).

In the last study [28] the mean forward displacement of the maxilla (vertical point A) was 3.40 ± 1.07 mm in MP/FM group and 2.80 ± 0.79 mm in FM group. The mandible showed backward rotation in both groups (vertical point B: MP/FM -1.00 ± 1.15 mm, FM -0.80 ± 2.15 mm).

A clockwise rotation of the mandible with an opening of the mandibular angle was highlighted in the subjects of the group treated with facemask in six studies [20, 21, 25–28]. This aspect was detected, despite being of minor importance, also in the group treated with skeletal anchorage in three studies [20, 27, 28]. On the other hand, two studies [21, 24] reported a minimal closure of the mandibular angle in the group with skeletal anchorage. Soft tissues have also been considered in some of the studies included in this review. Specifically, an improvement in the profile at the end of the treatment of both groups was noted in four studies [20, 22, 27, 28]. The remaining articles [21, 23–26] did not consider this parameter. All studies agree that, according to the results obtained, the correction of the Class III malocclusion by the use of skeletal anchors leads to a greater skeletal response compared to the use of a simple facial mask and reduces or eliminates the undesired dentoalveolar effects such as the excessive proclination of the upper incisors and the extrusion and the mesialization of the upper molars.

### 3.3. Quality of the Studies

The analysis of the quality of the studies is shown in Table 4. The results showed that only one study had a high quality [20], four medium/high [21–23, 27], three medium [24–26], and one low [28]. Sample description was partial in only one study [28]. Prospective enrolment was clearly reported in five studies [20–23, 27]. For diagnosis, Class III description and maturational stage distribution were full in eight [20–23, 25–28] and one [20] studies, respectively. For treatment, description was partial in three studies [24, 26, 28]. Withdrawals were declared in only two studies [20, 22]. Method error analysis was not included in only one study [28]. Blinding of measurements was followed in one study [26]. Statistical analysis was judged to be adequate in seven studies [20–24, 27, 28]. Finally, a previous estimate of sample size was not present in all studies.

## 4. Discussion

Analyzing the data collected by the various studies and the results obtained from the present research, it is possible to make some considerations with the aim of understanding which is the best operative protocol in the orthopedic correction of the skeletal Class III. Maxillary traction using a facemask is the most commonly used method for the resolution of Class III malocclusion [3]. This treatment produces benefits at the skeletal level but also undesirable dentoalveolar effects, such as the proclination of the upper incisors and the mesialization and extrusion of the upper molars [6, 7]. Therefore, in recent years, to increase skeletal effects and decrease dental effects, some clinicians and researchers have tried to transfer the orthopedic force directly to the bone through the use of temporary anchor devices (TADs) [14–17]. Although these systems obtain a better skeletal response, they present some unfavorable aspects. The placement of miniplates requires surgery for both insertion and removal, and some components may not be stable throughout the treatment [31]. Furthermore, there may be inflammation and irritation of the tissues in contact with the miniscrews. Another aspect to be taken into consideration is the limitation in the choice of the method to be used based on the age and the dentition phase of the patients. For placement of the miniscrews between the first molar and the second upper premolar the latter must completely erupt or, at least, have begun its eruptive path. The same thing happens in the placement of the mandibular plates that require the presence of the permanent canine in the arch and in the placement of the miniplates on the lateral nasal walls because they could interfere with the eruption of permanent canines if positioned at an early age. All the authors agree that the facemask is a practical and simple tool to obtain a maxillary protraction and it is recommended to perform the treatment at an early age (before 10 years) because the maxillary sutures have less resistance to orthopedic forces [3, 32] and, over the years, the skeletal effects decrease and increase, instead, the dental effects. The dental anchor prevents the total transfer of the orthopedic force directly onto the sutures because a large portion of the force is dissipated on the surrounding periodontal ligament and on the teeth. The improvement of the profile and the achievement of a positive overjet are obtained by a combination given by the proclination of the upper incisors and by the retrusion of the lower ones. On the other hand, maxillary traction with TADs results to be orthopedically effective even in patients aged between 10 and 12 years [16, 31, 32]. One possible explanation lies in the fact that the orthopedic force acts directly on the surrounding sutures, thus increasing the skeletal effect and eliminating the dental compensations. Comparing the two methods, almost all studies indicated a greater maxillary advancement in the group treated with skeletal anchorage. The results are in line with the scientific evidence present in the literature. Some authors, as Mermigos et al. (1990), Baik (1995), Ngan et al. (1996), Arman et al. (2006), Nartallo-Turley and Turley (1998), reported a forward movement of point A, respectively, of 1.76 mm, 2 mm, 1.9 mm, 2.11 mm, and 3.34 mm with the facemask, while Singer et al. (2000), Enacar et al. (2003), Kircelli et al. (2008) found a displacement of 4 mm, 4 mm, and 4.8 mm respectively by the used of skeletal anchored systems. Only two studies did not notice significant differences between the two groups [22, 26]. As regards the first, the probable reason could lie in the difference in force exerted since the force used in the MS / FM group was 250g per side, while in the FM group was 500g per side.

The number of included studies did not allow a full comparison of the effects produced by a maxillary expansion in comparison with MP alone (and in combination with the use of skeletal anchorage). Similarly, the short term of the available studies and the retrospective nature of several of them (Table 2) represent a further limitation of the present review, which also preventing the execution of a meta-analysis.

### 4.1. Clinical Implications

Considering the mixed effects (partly skeletal and partly dentoalveolar) that characterize the treatments with facemask, these find their best therapeutic usefulness at an early age to make the most of skeletal growth and reduce as far as possible the dental effects. Therapies using skeletal anchorage devices can be considered an effective treatment alternative to achieve maximum skeletal effects and minimal dental effects in patients with severe maxillary retrusion or early loss of deciduous dental elements since the use of the mask alone would cause more proclination of the maxillary incisors and mesialization of the molars with the closure of the spaces. In the subjects near the pubertal peak, it is recommended to use TADs to try to exploit the residual maxillary growth and not to solve the malocclusion with only dental compensations. The presence of skeletal anchors can be exploited at a later time to distalize the upper molars and/or increase the space in the maxillary arch during the fixed therapy phase. The BAMP method is more invasive than the mask, but the use of intraoral elastics is more comfortable and aesthetic and elastic traction can be active 24 hours a day. The clockwise rotation of the mandible is significantly reduced in therapies with TADs. Long-term effects of BAMP, although better than those obtained by MP alone, may still be not enough to treat successfully all the patients. More specifically, it is still not proven that BAMP may successfully treat those unstable patients in the long term.

## 5. Conclusions

- Facemask therapies induce a correction of skeletal Class III malocclusion through a combination of skeletal and dentoalveolar effects.
- Therapies with skeletal anchorage produce greater maxillary protraction reducing undesirable dental effects.
- In both groups, the best skeletal effects occur in prepubertal age but in the group with skeletal anchorage, responses are obtained even near the pubertal peak.
- The results of skeletal anchor devices will need to be verified with more randomized clinical trials and long-term follow-up.

## Figures and Tables

**Table 1:** Quality analysis of the included studies.

Pre-established Characteristics	Score
1. Adequacy of sample selection description based on age and sex across the groups	Full: 2 points; partial: 1 point
2. Study design for the inclusion of the treated group	Prospective: 1 point; retrospective or not declared: 0 points
3. Description of the Class III (full, skeletal, and/or dental parameters; partial, only dental parameters)	Full: 2 points; partial: 1 point
4. Distribution of the different maturational stages among the investigated subjects	Full: 2 points; partial: 1 point
5. Adequacy of treatment description based on three criteria: (a) orthodontic appliance; (b) description of TADs and their placement (miniscrews, miniplates); (c) treatment duration	Full: 2 points; partial: 1 point
6. Withdrawals declared or derivable	No/yes: 1 point; not declared: 0 points
7. Description of the method error analysis	Yes: 2 points; no: 0 points
8. Blinding for measurements	Yes: 1 point; no: 0 points
9. Adequacy of statistics based on the comparisons of the intragroup changes over time among/between groups (yes, when parametric or nonparametric tests used where appropriate; no, when parametric tests used when nonparametric tests would be more appropriate, multiple comparisons with uncorrected P values, statistical analysis only partially described)	Yes: 2 points, no: 1 points
10. Prior estimation of sample size or a posteriori power analysis	Yes: 1 point, no: 0 points

**Table 2:** Designs of the included studies.

**Author (year) **[reference]	**Study design**	**N(dropouts) Ca(cases) Co(controls) M(n), F(n) Mean Age**	**Class III Description**	**Skeletal maturation method/ stages**	**Ca (case group appliances) Co (control group appliances)**	**Treatment duration**	**Treatment Stopped**
Aglarci et al. (2016) [20]	CCT (prospective)	59(9) Ca(25) Co(25) M(Ca 12, Co 12), F(Ca 13, Co 13) MA(Ca 11.75 ± 1.23 y, Co 11.21 ± 1.32 y)	ANB° < 0 or Wits < 1; anterior crossbite	HWM /prepubertal (Ca 12 MP3 =, 13 S; Co 16 MP3 = 7 S, 2 PP2 =)	Ca (BAMP + class III elastics with a force of 200g for each side) Co (FM applied by a bite plate with a force of 400g per side)	Ca 0.76 ± 0.09 y, Co 0.52 ± 0.09 y	Ovj > 2mm (FM); Ovj > 0 (BAMP)

Cevidanes et al. (2010) [21]	CCT (prospective)	55(-) Ca(21) Co(34) M(Ca 10, Co 14), F(Ca 11, Co 20) MA(Ca 11.10 ± 1.10 y, Co 8.3 ± 1.10 y)	Wits < -1; anterior crossbite or incisor end- to-end relationship; class III molar relationship	CVM / CS1- CS3	Ca (BAMP + class III elastics with a force of 250g for each side) Co (RPE+FM with a force of 500g per side)	Ca 1y, Co 10m	Ovj > 0; II class molar relationship

Ge et al. (2012) [22]	RCT	49(6) Ca(25) Co(24) M(Ca 11, Co 12), F(Ca 14, Co 12) MA(Ca 10y4m, Co 10y6m)	ANB° < 0; Wits < -2; anterior crossbite	CVM / CS1- CS3	Ca (MS+FM with a force of 250g for each side) Co (RPE+FM with a force of 500g per side)	Ca 11m, Co 1y1m	Ovj > 0

Hino et al. (2013) [23]	CCT (prospective)	46(-) Ca(25) Co(21) M(Ca 12, Co 5), F(Ca 13, Co 16) MA(Ca 11.9 ± 1.8y, Co 8.1 ± 1.5y)	Wits < -1; anterior crossbite or incisor end- to-end relationship; class III molar relationship	CVM / CS1- CS3	Ca (BAMP + class III elastics with a force of 250g for each side) Co (RPE+FM with a force of 800g per side)	Ca 1.2 ± 1y, Co 10.1 ± 2.2m	Ovj > 0; II class molar relationship

Koh et al. (2014) [24]	CCT (retrospective)	47(-) Ca(19) Co(28) M(Ca 8, Co 7), F(Ca 11, Co 21) MA(Ca 11.21y, Co 10.09y)	OVJ < -2	CVM / CS3- CS4	Ca (MP+FM with a force of 400-500g for each side) Co (RPE+FM)	-	-

Lee et al. (2012) [25]	CCT (retrospective)	20(-) Ca(10) Co(10) M(Ca 5, Co 4), F(Ca 5, Co 6) MA(Ca 11.2 ± 1.2y, Co 10.7 ± 1.3y)	SNA°< 80°; ANB°< -1; A to N perp < 0; anterior crossbite	CVM / CS2 - CS4	Ca (MP+FM with a force of 400g for each side) Co (RPE+FM with a force of 400g per side)	Ca 1.0 ± 0.1y, Co 1.1 ± 0.1y	Ovj > 0; II class molar relationship

Ngan et al. (2015) [26]	CCT (retrospective)	60(-) G1(20) G2(20) G3(20) M(G1 8, G2 8), F(G1 12, G2 12) MA(G1 9.8 ± 1.6y, G2 9.6 ± 1.2y)	Wits < -3; ANB° < -2; anterior crossbite or incisor end- to-end relationship; class III molar relationship	-	G1 (RPE+FM with a force of 380g per side) G2(bone- borne hybrid hyrax RPE+FM with a force of 380g per side) G3 (untreated)	-	-

Sar et al. (2011) [27]	CCT (prospective)	45(-) G1(15) G2(15) G3(15) M(G1 10, G2 8, G3 7), F(G1 5, G2 7, G3 8) MA(G1 10.91y, G2 10.31y, G3 10.05y)	ANB°< 0; Nperp-A< 1; Wits < -2; anterior crossbite; class III molar relationship	HWM / PP2= -MP3	G1 (RPE+MP+FM with a force of 400g per side) G2 (RME+FM with a force of 400g per side) G3 (untreated)	G1 0.56 ± 0.16y, G2 0.78 ± 0.26y	Ovj > 4mm

Tripathi et al. (2016) [28]	CCT (retrospective)	20(-) Ca(10) Co(10) M(Ca -, Co -), F(Ca -, Co -) MA(Ca 10.10 ± 1.1y, Co 9.90 ± 1.1y)	ANB° < 0; anterior crossbite or incisor end- to-end relationship	CVM / CS1- CS3	Ca (RPE+MP+FM with a force of 400g per side) Co (RPE+FM with a force of 400g per side)	Ca 5.8m, Co 10m	Ovj > 4mm

**RCT **, randomized clinical trial; **CCT **, controlled clinical trial; **M **, males; **F **, females; **G **, group; **HWM **, hand-and-wrist maturation; **CVM **, cervical vertebral maturation; **CS **, CVM stage; **BAMP **, bone-anchored maxillary protraction; **FM **, facemask; **RPE **, rapid palatal expander; **MS **, miniscrews; **MP **, miniplates**; y **, years; **m **, months; **Ovj **, Overjet.

**Table 3:** Treatment effects in the included studies.

**Study**	**Dentoalveolar effects**	**Skeletal effects**	**Soft tissue effetcs**	**Clinical implications**
Aglarci et al. (2016)	Protrusion of the maxillary incisors in theboth groups, but in the FM group maxillary incisors measurements were at least double those of the SA group. Lower incisor retrusion in the FM group, while lower incisor protrusion was observed in the SA group.	Statistically significant increase in sagittal movement of points A and ANS in SA group as compared to FM group. Significant downward and backward rotation of the mandible in both groups, and it was more evident in the FM group. The increase in the occlusal plane angle was more prominent in the FM group.	Improvements in the soft tissue profile were achieved in both groups. Upper lip forward movement was obtained in both treatment groups.	Patients treated with mini- implants and mini-plates exhibited skeletal improvements, with little effect on mandibular position. The undesirable effects associated with FM treatment were eliminated with the SA method.

Cevidanes et al. (2010)	Lower incisor retrusion in the RPE/FM group, while lower incisor protrusion was observed in the BAMP group.	The BAMP protocol induces a significantly larger short-term response in terms of maxillary advancement and changes in midfacial length compared with the RPE/FM protocol. Slight mandibular counterclockwise rotation in BAMP group and clockwise rotation in FM group.	Not reported	The BAMP protocol induces significantly larger maxillary advancement than the RPE/FM therapy. Mandibular sagittal changes are similar, while vertical changes are better controlled with BAMP. Other favorable aspects of BAMP treatment are represented by the lack of clockwise rotation of the mandible and of retroclination of the lower incisors.

Ge et al. (2012)	Lingual inclination of the mandibular incisors was observed in both groups. Significant proclination of maxillary incisors only in the RPE/FM group.	Similar changes in maxillary advancement in both groups.	Improvements in the soft tissue profile were achieved. Upper lip forward movement was obtained in both treatment groups.	Compared with the RPE/FM protocol, the MS/FM therapy produces a similar maxillary advancement. The MS/FM protocol improves skeletal relationships and soft tissue profile and eliminates the undesired proclination of the maxillary incisors and reduces the mesialization of the maxillary dentition, which is present in the RPE/FM therapy.

Hino et al. (2013)	Comparing the 3D displacement of the maxillary incisors in the BAMP and RPE/FM groups, on average, the maxillary incisors were displaced forward by similar amounts.	The mean difference in displacement of the midface between the groups was approximately 1 mm.	Not reported	Orthopedic changes can be obtained with both RPE/FM and BAMP treatments.

Koh et al. (2014)	In the MP/FM group, the angulation of the maxillary incisors was retroclined at CVM3 compared to CVM4	MP/FM group showed closure of the mandibular plane with a high level of significance compared to that of the RPE/FM group. MP/FM produced a significant increase in the antero-posterior position of orbitale (SNOr) and A point.	Not reported	MP/FM produced significant increases in all measured anterior-posterior dimensions. MP/FM in CVM3 resulted in greater anterior-posterior changes, compared to RPE/FM, Statistically significant advantages of the MP/FM group in younger maturity and high vertical skeletal pattern compared with the RPE/FM group.

Lee et al. (2012)	MP/FM therapy induced less labioversion of the maxillary incisors compared with RPE/FM therapy.	The MP/FM group presented with greater forward movement of the maxilla than the RPE/FM group. The RPE/FM group exhibited a greater opening rotation of the mandible than the MP/FM group.	Not reported	MP/FM therapy induced a remarkable advancement of the maxilla, less posterior repositioning and opening rotation of the mandible, and less proclination of the maxillary incisors than RPE/FM therapy.

Ngan et al. (2015)	Greater proclination of the upper incisors in the RPE/FM group compared to the bone-anchored group.	Greater downward movement of the maxilla in the RPE/FM group compared to the bone-anchored group. Mandibular plane angle was found to open significantly more in the RPE/FM compared to the other group.	Not reported	Hybrid Hyrax RPE minimized the side effects encounter by tooth-borne RPE for maxillary protraction such as excessive forward movement of the maxillary molars and incisors, downward movement of the maxilla, and clockwise rotation of the mandible.

Sar et al. (2011)	Retrusion of the maxillary incisors in the MP/FM group and proclination in the FM group. Retrusion of the mandibular incisors in both groups.	Posterior rotation of the mandible in the MP/FM and the FM groups, whereas the rotation in the FM group was more evident. Lower and total facial heights increased in both groups, whereas greater changes were seen in the FM group compared with the MP/FM group. The maxilla advancement was more evident in the MP/FM group than in the FM group.	Improvements in the soft tissue profile in both groups.	The undesired dentoalveolar effects of conventional facemask therapies, such as mesialization and proclination of the maxillary teeth and extrusion of the maxillary molars, were reduced or eliminated with miniplate anchorage placed laterally to the apertura piriformis on both sides of the maxilla.

Tripathi et al. (2016)	Greater proclination of the upper incisors in the FM group compared to the MP/FM group. Slight retroclination of the mandibular incisors in both groups. Mesial movement of the maxillary molars in the FM group.	A greater forward displacement of the maxilla in the MP/FM group compared with the FM group. Downward and backward rotation of the mandible in both groups with more rotation in FM group.	Improvements in the soft tissue profile followed the underlying skeletal components in both groups.	Class III correction by MP/FM has significant skeletal effects with minimal dentoalveolar effects.

**Table 4:** Quality analysis of the included studies.

**Study**	**Sample description**	**Prospective enrolment**	**Class III description**	**Maturational Stages distribution description**	**Treatment description**	**Withdrawals**	**Method Error**	**Blinding for measurements**	**Adequacy of statistics**	**Prior estimate of sample size**	**Quality score**	**Judged quality standard**
Aglarci et al (2016)	Full	Yes	Full	Full	Full	Yes	Yes	No	Yes	No	14	High
Cevidanes et al. (2010)	Full	Yes	Full	Partial	Full	Not declared	Yes	No	Yes	No	12	Medium/ High
Ge et al. (2012)	Full	Yes	Full	Partial	Full	Yes	Yes	No	Yes	No	13	Medium/ High
Hino et al (2013)	Full	Yes	Full	Partial	Full	Not declared	Yes	No	Yes	No	12	Medium/ High
Koh et al (2014)	Full	No	Partial	Partial	Partial	Not declared	Yes	No	Yes	No	9	Medium
Lee et al (2012)	Full	No	Full	Partial	Full	Not declared	Yes	No	No	No	10	Medium
Ngan et al (2015)	Full	No	Full	Partial	Partial	Not declared	Yes	Yes	No	No	10	Medium
Sar et al (2011)	Full	Yes	Full	Partial	Full	Not declared	Yes	No	Yes	No	12	Medium/ High
Tripathi et al (2016)	Partial	No	Full	Partial	Partial	Not declared	No	No	Yes	No	7	Low
